# GOODD, a global dataset of more than 38,000 georeferenced dams

**DOI:** 10.1038/s41597-020-0362-5

**Published:** 2020-01-21

**Authors:** Mark Mulligan, Arnout van Soesbergen, Leonardo Sáenz

**Affiliations:** 1 Department of Geography, King’s College London, London, UK; 2 Environmental Engineering, Michigan Technological University (MTU), Houghton, USA

**Keywords:** Animal migration, Hydrology, Environmental impact

## Abstract

By presenting the most comprehensive GlObal geOreferenced Database of Dams to date containing more than 38,000 dams as well as their associated catchments, we enable new and improved global analyses of the impact of dams on society and environment and the impact of environmental change (for example land use and climate change) on the catchments of dams. This paper presents the development of the global database through systematic digitisation of satellite imagery globally by a small team and highlights the various approaches to bias estimation and to validation of the data. The following datasets are provided (a) raw digitised coordinates for the location of dam walls (that may be useful for example in machine learning approaches to dam identification from imagery), (b) a global vector file of the watershed for each dam.

## Abstract (summary)

**Table:** 

Measurement(s)	watershed • drainage basin • size • geographic location
Technology Type(s)	computational modeling technique
Factor Type(s)	location of dam
Sample Characteristic - Environment	dam
Sample Characteristic - Location	North America • South America • Africa • Europe • Asia • Australasia

Machine-accessible metadata file describing the reported data: 10.6084/m9.figshare.10538486

## Background & Summary

Dams and their reservoirs play an important role in social and economic development as they help supply seasonal water needs or generate renewable energy. Whilst dams and impoundments have been built for thousands of years, most large dams (defined as having a wall higher than 15 metres) have been built in the last 60 years and according to current estimates there are now around 58,000 of these large dams worldwide (https://www.icold-cigb.org/GB/world_register/general_synthesis.asp) with an estimated cumulative storage capacity of between 7,000–8,300 km^3^ ^1,2^. This storage is equivalent to one-sixth of the total annual river flow into the oceans^3^. In addition, there are likely many more small dams and impoundments, as yet unrecorded by the global databases. No reliable figures exist on the numbers of these but a study by Lehner *et al*.^4^ estimates there may be more than 16 million smaller impoundments with surface area larger than 100 m^2^ with a combined surface area of around 306 000 km^2^ increasing the Earth’s natural terrestrial freshwater surface by more than 7%.

Nearly 50% of the world’s large dams were built primarily for irrigation, estimated to directly contribute water that supports 12–16% of global food production^5^. With a growing population, and food demand estimated to rise by 70% by 2050^6^, more dams and storage reservoirs will be required to support increased irrigation. Furthermore, hydropower contributes around 70% of the world’s renewable electricity production which in turn currently makes up 24% of global electricity production^7^. However, it is estimated that only 22% of the world’s technically feasible hydropower potential is currently exploited^8^. With an expected rise in energy demand of 56% between 2010 and 2040^8^, and the need for more of this to be from renewable sources, it is likely that more and larger dams will be built in coming decades. The increasing demands for energy, water storage and flood control are particularly pertinent in low income countries, where fewer dams have been built to date, resulting in more and larger dams being planned and constructed in these areas^8,9^.

Despite the social and environmental concerns associated with dams^10–13^ and the recognized importance of accurate assessments of the role and impacts of single dams and downstream cascades of dams and reservoirs^5^, research has so far been limited by a lack of consistent data and assessment tools, particularly at transboundary and global scales. Globally consistent geo-referenced data on dams such as the GRanD database^4^ have only been available relatively recently. Containing 6,862 records (V1.1), this database still only captures around 12% of the total estimated 58,000 large dams, though it does provide detailed attribute data on the properties of each dam.

In this paper we address the need for more spatially comprehensive data by presenting the largest open source global geo-referenced database of dams to date containing more than 38,000 georeferenced dams as well as derived data on their associated catchments. Unlike GRanD, we do not provide detailed attribute data on the characteristics of each dam and reservoir. We focus on dams with concrete walls, observable in global satellite imagery from LANDSAT (15 m), IKONOS (<1 m) and SPOT (2.5 metres) and capture both the large dams of the existing global assessments from GRanD^4^ and ICOLD (http://www.icold-cigb.org) but also medium sized dams, though not small agricultural reservoirs. We capture these data using a globally consistent methodology that avoids the spatial bias of crowdsourced datasets or of aggregated national datasets. We call this the GOODD (GlObal GeOreferenced Database of Dams). Dams were identified by examining global water bodies datasets systematically, 1-degree tile by tile across the world and identifying reservoirs in underlying Google Earth imagery. From the reservoir the dam wall was then identified and the location of exit of the dammed river at the wall digitised as a latitude and longitude representing the dam location. GOODD mostly contains reservoir dams with only a few run-of-river dams that are found for example in the Mekong river basin. Due to the lack of reservoir these dams are less easy to identify in satellite imagery but are also less relevant in terms of water storage and river fragmentation.

This database can be used for a wide range of assessments into the functioning and impacts of dams. We focus on identifying the catchments supplying water (and sediment) to these dams. Understanding these catchments is important to understanding the impacts of climate change, land use change and land management interventions supporting ecosystem service provision in the watershed of these points at which those ecosystem services are realised as stored water or hydropower energy. We describe the development and validation of GOODD and highlight some remaining biases of the data.

## Methods

### Digitising global dams

Dams were digitised by scanning through one by one degree tiles on the Google Earth geobrowser (http://earth.google.com) using a so-called GeoWiki coded in KML (Keyhole Markup Language) and linked to an online database. The GeoWiki allows multiple users to add to the database without interfering with each other’s efforts and provides a clear indication of which areas are complete and which are left to scan. Most of the digitising was carried out between 2007–2011 with additional updates in 2016. The 2011 database was updated for each continent using updated national registers for India for 2015 (http://cwc.gov.in/national-register-large-dams) and the 2016 US National Inventory of Dams database (http://nid.usace.army.mil/). Other sources used were the spreadsheet of major dams in China developed by International Rivers (https://www.internationalrivers.org/resources/spreadsheet-of-major-dams-in-china-7743) and other web based sources for individual countries. Finally, a cross-check with the GranD v1.1^4^ database was made and the few dams not already included in GOODD were added from the Google Earth imagery. The combined 2016 updates resulted in the addition of 780 dams in North America, 190 dams in South America, 418 dams in Europe, 868 dams in Asia, 211 dams in Africa and 89 dams in Oceania.

To be able to identify likely locations of dams based on their reservoirs, a Google Earth visualisation of the Shuttle Radar Topography Mission (SRTM) Water Body Dataset (SWBD) (http://geodata.policysupport.org/swbd) was used as a guide in the digitising process. Each digitised dam wall location was saved as a precise latitude and longitude and was attributed a unique ID number.

### Minimum dam size criteria

Since Google Earth imagery resolution differs for different areas around the world and at different periods in time, particularly for the period in which most of the digitising was carried out, a minimum dam size for inclusion in the dam database was established, defined as the minimum size that could still be identified as a dam with low resolution (LANDSAT Geocover 2000) resolution imagery. A pilot with 100 dams from the US National Inventory of Dams database showed that a reservoir length of 500 metres and dam wall length of 150 metres for small dams could be identified with certainty from this low resolution imagery available globally in Google Earth.

### Supporting sources

A number of supporting sources were used to verify and validate the catchments of the dams. These sources included geo-referenced digital information, lists and printed documentation. Included in the GEOWIKI on Google Earth are the SRTM SWBD and the US National Geospatial-intelligence Agency GEONET Names Server (NGA GNS) Gazetteer place names database processed for Google Earth by Mulligan (http://geodata.policysupport.org/places). GNS is a geographic feature database containing at the time more than 4 million features globally. Once the globe had been covered for digitising systematically tile by tile, a number of databases were used to help identify significant but missed dams, for example obscured by cloud cover in the Google Earth imagery, which was more common during the period 2007–2010 than it is now. Information from the World Register of Dams (WRD) of the International Commission On Large Dams (ICOLD)^14^ was used to identify dams more easily in a few areas with low resolution imagery on Google Earth (e.g. Russia) by matching place names from the Gazetteer with the location attribute listed in the ICOLD database such as nearest village or the name of a dam and then looking for the reservoir. The NID geo-referenced database by the US Army Corps of Engineers (http://nid.usace.army.mil/). was used as an overlay in Google Earth to help locate dams in the USA. Similarly, the FAO AQUASTAT (http://www.fao.org/nr/water/aquastat/main/index.stm) database was used to locate dams on the African continent. Other sources that were used included information from the World Commission on Dams^5^, the Brazilian Committee on Dams (http://www.cbdb.org.br/5-69/Cadastro%20Nacional%20de%20Barragens), the Venezuelan Committee on Dams (http://www.covenpre.org.ve/), the Mekong River Commission main streams dam map^15^, the Global Lakes and Wetlands Database^16^, the National Register of Large Dams in India for the years 2009, 2015 and 2018 (http://cwc.gov.in/national-register-large-dams), The International Rivers web pages and the Spanish Association on Dams and Reservoirs for the year 2010 (http://www.seprem.es/). Finally, in the 2016 update, the dataset was cross-checked with the GRanD^4^ dataset. Data from many of these sources were also used to validate the calculated dam catchment areas against those presented within these databases.

### Production of watersheds

Contributing areas of the dams were calculated by manually snapping the dam locations to a hydrologically correct streamflow network derived from the HydroSHEDS^17^ Digital Elevation Model (DEM) at 30-arc second resolution. As the Hydrosheds SRTM based DEM product only covers the globe between latitudes of 60 degrees south and 60 degrees north, the global Hydro1K DEM at 30-arc second resolution was used for the land masses in the most northern regions (Scandinavia, North Russia, Canada, Alaska), where there are fewer dams. The DEM’s were mosaicked using Arcmap 9.3 GIS software^18^ and a set of 6 continental tiles with overlapping extents was produced (Table 1) that were used to derive stream flow networks using a D8 algorithm^19^.

**Table 1:** Details of continental DEM tiles. These tiles were used to derive the hydrologically correct streamflow networks for snapping of dams.

Continent	Resolution	Latitude		Longitude	
		From	To	From	To
North America	30 arc second	90 North	0 North	−180 West	−50 West
South America	30 arc second	20 North	−60 South	−90 West	−30 West
Africa	30 arc second	−40 South	40 North	−20 West	60 East
Europe	30 arc second	90 North	10 North	−30 West	70 East
Asia	30 arc second	80 North	0 South	50 East	180 East
Australasia	30 arc second	30 North	−30 South	−180 West	−130 West

Stream flow networks were derived using the D8 algorithm in the PCRaster Environmental Modelling Language^20^. We used PCRaster derived stream flow networks and not the stream flow networks that are part of the HydroSHEDS distribution to ensure consistency with earlier pre-Hydrosheds derived stream flow networks used for the pantropical part of the dam database produced in 2007 as well as to ensure a consistent approach to the derivation of a stream flow network for the areas above 60 degrees North, which HydroSHEDS does not cover. This pre-hydroSHEDS flow network was also used by version 1 of the WaterWorld model^21^ that the database was produced to work with.

Local Drainage Direction (LDD) maps indicating the direction of flow of material (e.g. water or sediment) from one cell to its immediate steepest down slope neighbouring cell were calculated using the PCRaster *lddcreate* operator, using 1e31 as the parameter value for outflow depth, core volume, core area and catchment precipitation in order to remove all pits except those at river mouths.

### Verification and relocation on the river network

Due to the relatively coarse resolution (1-km) of the DEM-derived streamflow network representing rivers, the actual spatial location of a river and dam in satellite imagery does not always coincide with the location of the same river on this stream network. Therefore, in order to generate reliable catchments associated with the dams, a visual inspection was carried out for every dam to ‘snap’ it to the appropriate flow line, in cases where they did not already overlay.

### Calculation of catchment areas

Once all dams were accurately aligned with the stream flow network, the upstream catchment areas of dams were calculated using the PCRaster operator *subcatchmen*t applied to the dam locations. This function applies the unique ID of a pixel to all upstream pixels on a given flow network starting at the location of the dam in this case. Where dams are nested within the catchments of other dams, the subcatchment allocations are maintained. The continental maps of catchment areas were visually inspected in Google Earth in order to check if the derived catchments were of the expected size and shape. Validation was also done against published supporting sources (see technical validation). If a dams’ catchment was found to be erroneous (i.e. a smaller or larger catchment than expected), the snapped dam location was checked and if found in error moved to the correct location on the stream flow network and a new catchment map was calculated using the procedure above.

## Data Records

The GOODD database^22^ is available on the figshare repository. In addition, the database is available through an unrestricted data repository hosted by King’s College London accessible through the Global Dam Watch data portal (http://www.globaldamwatch.org). Both the point data of dam locations and the polygon data for the catchments are available as Esri Shapefiles using the World Geodetic System 1984 (WGS84) datum and geographic coordinate system. Both files are compressed into a single zipped file. Dam IDs match the corresponding catchment IDs in the attribute tables of the datasets. No other attribute data are provided except latitude and longitude of dam locations.

## Technical Validation

### Number of dams by continent and country

**Fig. 1:**
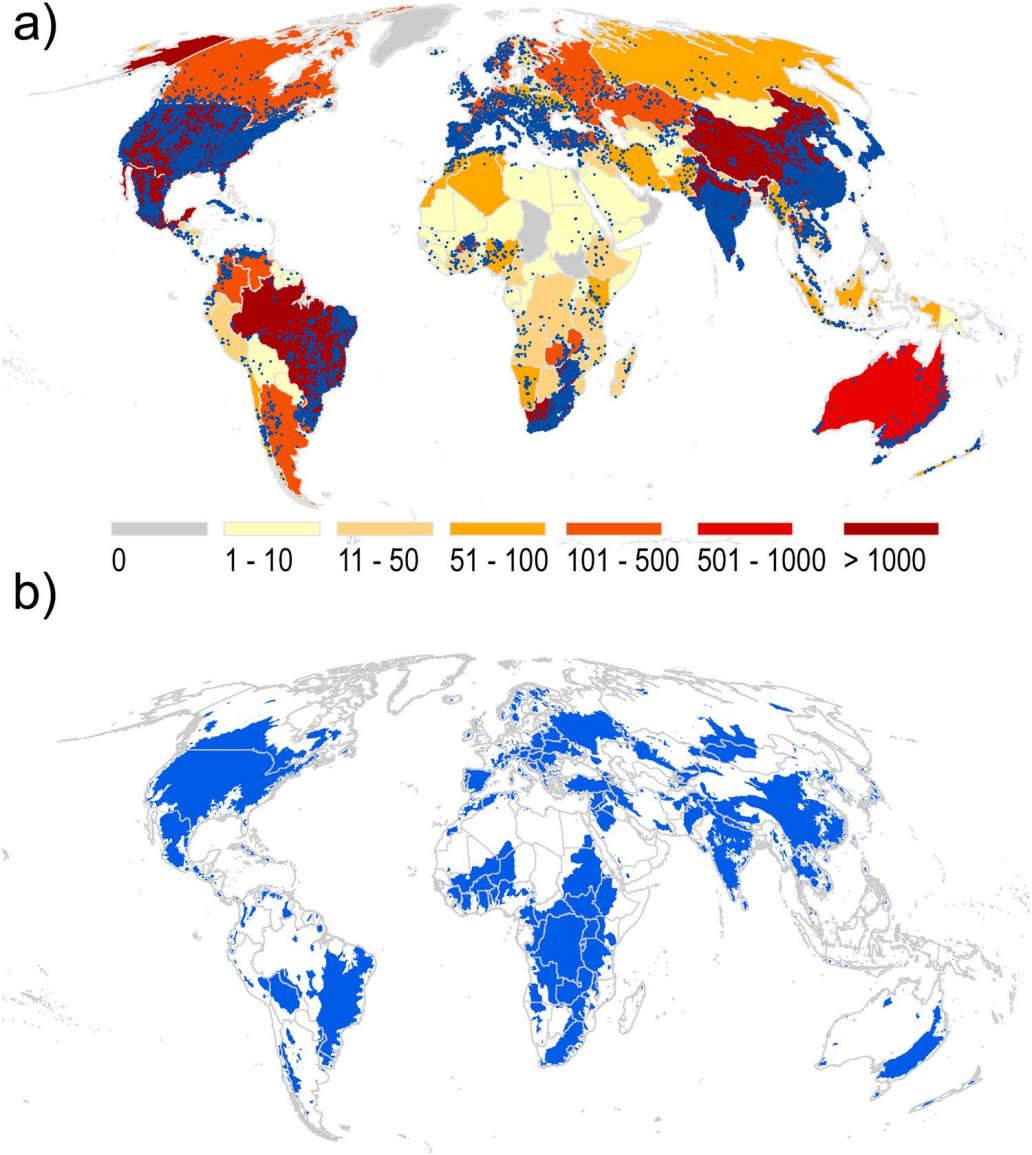
Dams and catchments in GOODD database. (**a**) Shows the number of dams in each country (yellow to red colours) and individual dam locations (blue dots) and (**b**) shows the area of terrestrial land draining into a dam in blue.

The database V1 holds 38,667 dams (Fig. 1a) with their associated catchments draining nearly 35% of global land area (excluding Antarctica) and around 32% of all tropical land area (Fig. 1b). On a continental basis, nearly 50% of all dams are found in Asia (18,951 dams), 7.1% in Europe (2,760 dams), 16.4% in North-America (6,359 dams), 16.5% in South-America (6,394 dams), 9.2% in Africa (3,558 dams) and 1.7% in Oceania (645).

The fewest dams per country are found on the African continent and the highest density is found in Asia, mostly within China and India, (9,215 and 6,785 large and medium sized dams respectively) along with Brazil (5,366), USA (4,602) and South-Africa (1,431).

The most recent edition of the World Register of Dams of ICOLD (2019), contains information on 57,985 large dams, defined as dams with a structural height above foundation not less than 15 metres. Whilst the WRD is considered to be the most comprehensive database on dams^23^ it relies on data being supplied by national committees and therefore data may be incomplete and inconsistent across the world, hence challenging for consistent global or transboundary analyses^14^. Moreover, many of the dams in this database are not georeferenced and therefore cannot be used to carry out spatial and hydrological analyses. The ICOLD database is only available dam by dam and is not freely available as a global database or open source.

The 2018 inventory of large dams in India by the National Register of Large dams (http://cwc.gov.in/national-register-large-dams) reports 5,264 large dams and another 437 dams under construction in India in 2018 (Table 2) which corresponds with the number of dams provided by ICOLD for this country. The higher number of dams in the GOODD database (6,785) is therefore likely to be caused by the inclusion of smaller dams which are omitted by both these registers. The Brazilian Committee of Dams (CBDB) reports directly to ICOLD and lists around 1,400 dams in 2014 (http://www.cbdb.org.br/5-69/Cadastro%20Nacional%20de%20Barragens). However, the CBDB acknowledges that data on many dams in Brazil is still incomplete, which could explain the large discrepancy with the number of dams identified by GOODD in Brazil (5,363). The South Africa list of registered dams for 2016 compiled by the Department of Water and Sanitation of the Republic of South Africa (http://www.dwa.gov.za/default.aspx) contains information on 5,226 dams of which 1,206 are considered to be medium to large in size. Therefore, the 1,432 dams in the GOODD database can be considered a good representation of large dams in South Africa. Good quality (geo-referenced) dam data for the United States is available from the National Inventory of Dams (NID) which reports 6,433 dams higher than 50 feet in 2013 (http://nid.usace.army.mil/). An earlier version of this dataset was used as ancillary data in the GOODD development process for the USA. The exclusion of industrial and mines tailings dams and recreational lake reservoirs in the NID database explains the discrepancy with the number of large dams reported by GOODD. The GOODD database captures around 40% of ICOLD reported dams in China. Independent georeferenced information on large dams in China is not available limiting the verification of this number. Most of the GOODD database was developed between 2008 and 2010 and therefore it is likely that some dams built after this date are not captured by it. Furthermore, low imagery resolution (15 m pixels) and poor image quality in some parts of China at the time of digitising is likely to be the cause of the underrepresentation of smaller Chinese dams in GOODD.

**Table 2:** Countries with most large dams in GOODD and compared with other data sources. The number of dams in the five countries with the most dams in GOODD are compared with reported numbers in the ICOLD^a^ database and other sources detailed underneath the table.

	GOODD	ICOLD	Other Sources
China	9,215	23,841	—
India	6,785	5,100	5,264^a^
Brazil	5,366	1,364	1,400^b^
United States	4,602	9265	6,433^c^
South Africa	1,431	1,112	1,206^d^

^a^Central Water Commission (2018). ^b^Brazilian Committee of Dams, (2014). ^c^US Army Corps of Engineers, (2013). ^d^Department of Water and Sanitation, Republic of South Africa (2016).

### Image resolution, cloud cover and contributors

At the time of digitising most of the database, the spatial resolution of imagery in Google Earth ranged from around 1–5 m (IKONOS, Quickbird, SPOT and aerial photography) to around 30 metres based on Landsat Geocover Mosaics from the period around year 2000. The higher resolution imagery was generally found in urban areas and high income countries whereas lower resolution imagery was found in low income countries and more remote areas such as northern Canada and Siberia. Since the visual identification of dams in imagery depends on the spatial resolution of the imagery, geographical variation in image spatial resolution could potentially lead to spatial biases in number of dams identified.

**Fig. 2:**
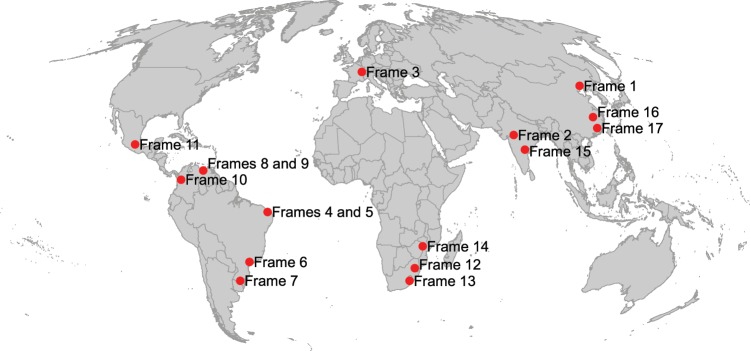
Location of validation frames. These seventeen 1-degree validation frames were used to assess the potential underestimation of the number of dams in areas with low resolution imagery.

To account for the possible underestimation of the number of dams in areas with low resolution imagery, a number of areas were digitised twice using the historical imagery feature of Google Earth (available from version 5.1). Seventeen 1-degree tiles (approx. 170,000 km^2^) previously digitised in high resolution imagery areas that contain dams were randomly selected for this procedure (Fig. 2). The tiles were re-digitised using older Landsat Geocover mosaic (circa 2000) imagery. The difference between both sets of dams was then used as an indicator of the potential number of dams that are not represented in the database for areas still under low resolution imagery at the time of database development. This procedure was applied in both high dam density areas and low dam density areas. Table 3 presents the results of this validation process for the 17 validation frames.

**Table 3:** Location and validation results for validation frames. These frames were used to assess the potential underestimation of the number of dams in areas with low resolution imagery. A map of locations is provided in Fig. 2.

Location	Country	Region	Nr. of dams identified from Landsat imagery	Nr. of dams identified from high resolution imagery (1 m)	% representation
Frame 1	China	East	7	9	77.7
Frame 2	India	Central	34	70	48.5
Frame 3	France	North East	2	10	20.0
Frame 4	Brazil	North East	12	17	70.6
Frame 5	Brazil	North East	24	27	88.9
Frame 6	Brazil	Central	16	28	57.1
Frame 7	Brazil	South East	87	112	77.7
Frame 8	Venezuela	North	7	8	87.5
Frame 9	Venezuela	North	17	20	85
Frame 10	Colombia	Central North	1	3	33.3
Frame 11	Mexico	Central	25	33	75.8
Frame 12	South Africa	Central North	28	34	82.4
Frame 13	South Africa	Central South	28	33	84.8
Frame 14	Zimbabwe	North East	80	100	80.0
Frame 15	India	Central	27	36	75.0
Frame 16	China	East	14	14	100
Frame 17	China	South East	68	82	82.9

The average underestimation due to low resolution imagery across the 17 validation frames is 27.8%. The extent to which this underestimation has led to underestimation in the global database is likely to be low as many of the areas with low resolution were generally confined to more remote and uninhabited areas that tend to have fewer dams, such as Siberia and North-Canada. Human modified landscapes that have most of the world’s dams tend to be covered by high resolution imagery. Moreover, some of the differences likely reflect the building of new dams between the capture dates of the low resolution and high resolution imageries.

Another source of potential error related to imagery derives from cloud cover. Some areas, particularly in the tropical mountains were obscured by heavy cloud cover in the native Google Earth imagery which makes it virtually impossible to locate a dam. To be able to find dams in cloud affected regions, Terrascope imagery (http://geodata.policysupport.org/terrascope) was used. Terrascope is a Google Earth implementation of the LANDSAT MSS, TM and ETM+ cloud free ortho mosaics for the 1970s, circa 1990 and circa 2000. In Terrascope, these images were converted to Google Earth superoverlays with resolutions ranging from 57 metres/pixel for the 1970s (MSS) to 14.25 metres/pixel for circa 2000 (based on ETM+). Particularly the 1990s imagery proved to be very useful in detecting dams under cloud affected imagery in Google Earth native imagery (based on Geocover 2000s at the time of digitising).

There are also potential differences in dam identification as different users may have different interpretations of what constitutes a dam or should be included in the database (see minimum size dam criteria). The majority of the database was assembled by the authors with only a limited number (~4%) of dams added by a small additional group of contributors. To be able to account for any potential differences between various contributors, a number of 1-degree tiles were digitised a second time by a different contributor, making sure the imagery used in the digitisation process was the same by looking at the date when the tile was finished and if necessary setting the imagery date on Google Earth to the imagery available at that time using Google Earth’s history feature.

On average more than 80% of dams were found by both contributors (Table 4), i.e. differences were less than 20%.

**Table 4:** Location of tiles and results of dam identification validation. These tiles were used to assess the potential differences in dam digitising by different contributors.

Country/Continent	LatLL	LongLL	Initial	Re-digitised	% overlap
China	30N	105E	242	212	87.6
India	19N	77E	94	90	87.6
Africa	26S	29E	93	66	70.9
South America	07S	50W	6	8	100.0
South America	06S	75W	5	3	60.0
North America	27N	100W	190	188	98.9
South America	10N	67W	89	55	61.8

Since most discrepancies are likely to be associated with the representation of smaller dams, whose watersheds are more likely to be found within the watersheds of larger dams, these errors will likely have little impact on the overall watershed statistics. Also the largest watersheds will tend to be dammed by the larger dams and therefore the majority of watersheds should be definable from digitizing the largest dams.

### Validation of catchment areas

**Fig. 3:**
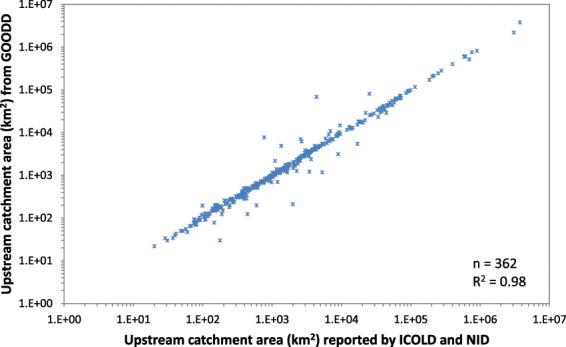
Validation of upstream catchment areas against reported data from ICOLD and NID. This figure shows the results of the validation of 3,562 GOODD calculated upstream catchment areas with ICOLD16 and NID (http://nid.usace.army.mil/) reported catchment areas for these dams.

To verify the accuracy of the calculated catchment areas, the derived area values were compared against reported figures available from the World Register of Dams (WRD)^14^ the Dams and Development Project (DDP)^24^, the Brazilian Committee on Dams (CBCD), Venezuelan Committee on Dams, the Mekong River Commission main streams dam map, the geo- referenced database of African dams (AQUASTAT) and the Global Lakes and Wetlands Database (GLWD)^16^. A random sample of 3,562 large dams for which catchment areas were available in the literature were compared against the calculated derived catchment areas yielding a good correlation between calculated catchment size and reported size for most of the dams (R^2^ = 0.98, Fig. 3). The average difference between calculated and reported catchment area amounts to 8.8%. However, some larger differences were found for specific dams. When looking into those specific cases, errors were found in the areas supplied by the supporting sources used for validation rather than those calculated from GOODD. This was the case for the La Miel dam in Colombia, where the reported catchment area based on ICOLD (2003)^14^ is almost ten times larger than the calculated area but also nearly eight times larger than the river basin in which the dam is located. Other such cases were also found for the La Honda dam in Venezuela and the Segredo and Embarracoda dams in Brazil.

## Data Availability

Arcmap 9.3 GIS software was used to mosaic the DEMs into continental tiles. PCRaster version 3 was used to derive the flow networks using standard operators. All spatial analyses were carried out using Arcmap 9.3.
